# Experience of Supporting Telemedicine Networks With the Collegium System: First 6 Years

**DOI:** 10.3389/fpubh.2019.00226

**Published:** 2019-08-21

**Authors:** Richard Wootton, Laurent Bonnardot

**Affiliations:** ^1^Norwegian Centre for Integrated Care and Telemedicine, University Hospital of North Norway, Tromsø, Norway; ^2^Fondation Médecins Sans Frontières, Paris, France; ^3^Department of Medical Ethics and Legal Medicine, Paris Descartes University, Paris, France

**Keywords:** telemedicine, low-resource settings, tele-expertise, education, humanitarian

## Abstract

The Collegium system was first made available in 2012 to support organizations conducting humanitarian or non-commercial telemedicine work in low resource settings. It provides the technical infrastructure necessary to establish a store-and-forward telemedicine service. During the subsequent 6 years a total of 46 networks were established, based on the Collegium infrastructure. The majority of the networks were set up to provide a clinical service (33), with six designed for education and training, and the remainder for test or administrative purposes. Of the potentially operational networks which were set up (i.e., those established for clinical or educational purposes), 15 networks (38%) were stillborn and did not handle a single case after being established. In contrast, the two most active networks had handled almost 12,000 cases. The average case rate of the five most active clinical networks operating in low-resource settings (i.e., the total number of cases divided by the length of time for which the network had been established) ranged from 0.5 to 29.4 cases/week. Across the networks there was little evidence of sigmoidal growth in activity, which is consistent with reports of other telemedicine activity in North America. A brief survey was sent to 49 network coordinators, from 31 networks. Responses were received from 9 coordinators (18% of those invited to participate). The median satisfaction with the system was 8 (on a scale from 1 = not at all satisfied to 10 = very satisfied). The free text comments were mainly technical suggestions regarding image transfer, the mobile application, or other modes of communication. The results of operating the Collegium system demonstrate that supporting telemedicine work in low resource settings can be successful, since the networks handled a very wide range of clinical cases, and at activity levels up to several cases per day. However, approximately one-third of the networks that were established did not handle a single clinical case. Nonetheless, this might represent a form of success in the sense that it prevented the waste of resource involved in an organization purchasing a telemedicine infrastructure only to find that it was not used.

## Introduction

Collegium Telemedicus is a not-for-profit organization which provides the technical infrastructure necessary to establish a store-and-forward telemedicine service. The aim is to support organizations conducting humanitarian or non-commercial work in low resource settings. There is no charge for using the Collegium system if the telemedicine service is humanitarian in nature (see https://collegiumtelemedicus.org for further details). The Collegium system is provided under a Software As A Service model, and is designed to be easy to use (“usability”), with few technical (hardware and software) requirements for users, and to be able to serve requests from increasing numbers of users (“scalability”).

The Collegium system was first made available in November 2012 (1). Our hypothesis was that organizations delivering health care in low-resource settings would make use of it to establish trial telemedicine services. We expected that those which succeeded would then either convert to a bespoke service based on the Collegium model, or would transfer their operation to another telemedicine provider.

The aim of the present study was to verify or refute the hypothesis about the use of the system, and to examine the outcomes of the first 6 years of its use.

## Methods

Information for potential users of the system was made available via the web^1^. Each organization establishing a network on the Collegium system provided a prospectus describing the purpose of the proposed work, and nominated an individual as a sponsor or guarantor. All network guarantors provided consent to the use of anonymized data from their network for research purposes. Anonymized information about the nature of each network was extracted from its prospectus. This included:

- nature of the organization (informal, charity, other)
- country of the organization
- purpose of the work (clinical, educational, administrative/test)
- countries of the catchment area for cases
- clinical specialties for cases
- network languages (the Collegium system is available in English, French, Spanish, Arabic, and Portuguese).

In order to measure network activity, the numbers of clinical or educational cases (i.e., non-test cases) managed on each network were examined for the epoch November 2012 to October 2018, inclusive. A clinical case corresponded to a patient, and an educational case corresponded to a case report. Mean case rates were calculated from the date that each network was established until 31 October 2018, i.e., the total number of cases received in this period was divided by the number of days of operation and expressed as a mean rate (cases/week). For convenience, network activity was also summarized as:

- 0 = no activity (fewer than 10 clinical cases)
- 1 = some cases handled, but not active at the time of study
- 2 = active at the time of study.

The types of case actually handled in the networks, as opposed to the types of cases that were specified in the network prospectus, was examined with reference to the types of specialist involved in answering each case. Thus, if a particular case had been sent to a pediatrician and to a radiologist for reply, the case was categorized as requiring both pediatric and radiologic expertise.

Finally, a short survey (Appendix 1 in Supplementary Material) was sent by email to all network coordinators for whom there was a valid email address.

Statistical analyses were conducted using the R Framework via the Wessa interface (2).

## Results

At the end of the study period a total of 46 networks had been established, based on the Collegium infrastructure. The majority of the networks (33 or 72%) had been designed to provide a clinical service. Six networks (13%) had been designed to provide education or training. The other seven networks (15%) were used by Collegium for internal administrative purposes concerning the development of the system, e.g., software testing (see Table 1).

**Table 1:** Main purpose of the networks operating in the Collegium domain, and in private domains*.

**NETWORKS IN THE COLLEGIUM DOMAIN**			
**Test/Administrative**	**Clinical**	**Educational**	**Domain total**
3	32	6	41
**NETWORKS IN PRIVATE DOMAINS**			
**Test/Administrative**	**Clinical**	**Educational**	**Domain total**
4	1	0	5
**TOTAL NETWORKS**			
**Test/Administrative**	**Clinical**	**Educational**	**Grand total**
7	33	6	46

* *Private domains represent Collegium networks that are accessed via the URL belonging to the sponsoring organization, rather than through the Collegium home page at https://collegiumtelemedicus.org*.

### Characteristics

**Figure 1:**
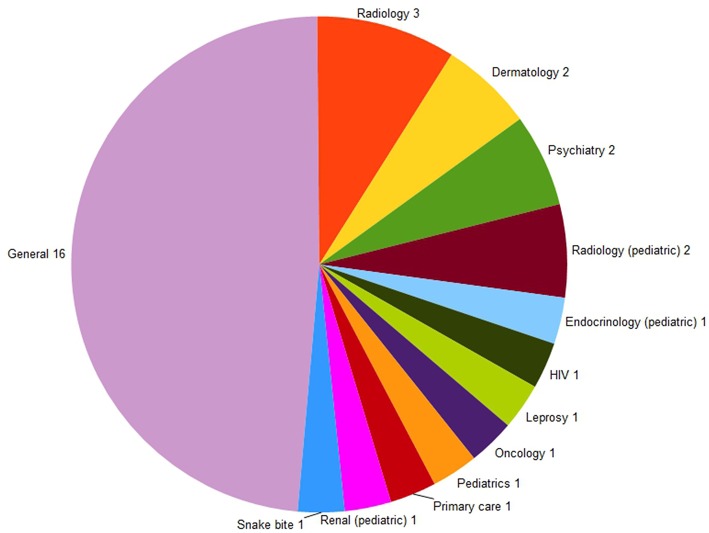
Types of cases that the clinical networks were established to manage.

There was wide variation in the characteristics of the networks and in the areas they were designed to serve (Table 2). Almost half of the clinical networks had been established in order to manage telemedicine cases of all specialties (surgical, medical, nursing, allied health). The other half of the networks had been established to manage single-specialty cases, such as radiology, dermatology, or psychiatry (Figure 1).

**Table 2:** Characteristics of the networks.

**Network ID**	**Purpose^a^**	**Organization^b^**	**Specialty^c^**	**Languages^d^**	**Sponsor^e^**	**Catchment^f^**	**Activity^g^**
0	Admin	–	–	–	–	–	–
4	Admin	–	–	–	–	–	–
9	Clin	Informal	Dermatology	En	USA	Ethiopia	0
11	Admin	–	–	–	–	–	–
12	Admin	–	–	–	–	–	–
13	Admin	–	–	–	–	–	–
14	Edu	Informal	Ultrasound	En	UK	Global	1
17	Edu	Informal	Nursing (pediatric)	En	Australia	Pacific	0
18	Clin	Informal	Dermatology	En	New Zealand	New Zealand	2
19	Clin	Other	HIV	En	Canada	Malawi	0
21	Admin	–	–	–	–	–	–
22	Clin	Charity	General	En; Fr; Sp	France	Global	2
23	Clin	Informal	Pediatrics	En	Nigeria	Nigeria	0
24	Clin	Charity	General	En	Switzerland	Global	0
25	Clin	Charity	Radiology (pediatric)	En; Sp	USA	Global	2
26	Clin	Informal	Psychiatry	En; Ar	USA	Syria	1
27	Clin	Informal	General	Fr; En	France	France	0
28	Admin	–	–	–	–	–	–
29	Clin	Informal	General	En; Ar	Yemen	Yemen	0
31	Clin	Informal	Oncology	En	UK	Uganda	0
32	Clin	Informal	Snake bite	En; Fr	France	Africa	0
33	Clin	Informal	Endocrinology (pediatric)	En; Fr; Sp	Canada	Haiti	2
34	Edu	Informal	Tuberculosis	En	Australia	PNG	2
35	Edu	Informal	Epilepsy	En	USA	Grenada	0
36	Clin	Informal	General	En	USA	Haiti	0
37	Clin	Informal	Radiology (pediatric)	En	UK	Nepal	0
40	Edu	Informal	Dermatology	En; Fr	France	Global	0
41	Clin	Informal	Radiology	En	USA	Cameroon	0
42	Clin	Charity	General	En	USA	Global	2
43	Clin	Informal	Renal (pediatric)	En; Fr	France	Africa	0
44	Clin	Other	General	En; Fr	France	Global	0
45	Clin	Informal	General	En	Canada	Guyana	2
46	Clin	Other	General	En; Fr	France	Africa	0
47	Clin	Informal	General	En; Fr	France	Africa	0
48	Clin	Informal	Psychiatry	En	USA	Somalia	0
49	Clin	Informal	General	En; Fr	France	Mali	0
50	Edu	Informal	Nursing	En	New Zealand	Global	0
51	Clin	Informal	General	En; Fr	France	Chad	0
52	Clin	Informal	Radiology	En	PNG	PNG	0
53	Clin	Informal	Radiology	En	Australia	Samoa	0
54	Clin	Informal	Primary care	En; Fr	USA	Cameroon	0
55	Clin	Informal	Leprosy	En; Fr, Sp, Pt	France	Global	0
56	Clin	Informal	General	En; Sp	USA	Honduras	0
57	Clin	Informal	General	En; Sp	USA	Peru	0
58	Clin	Other	General	En; Fr	France	Africa	0
59	Clin	Other	General	En	Tristan da Cunha	Tristan da Cunha	0

a *Purpose: Admin, administrative or test purposes; Edu, educational or training; Clin, clinical*.

b *Organization: type of organization. Informal/Charity (humanitarian organization)/Other*.

c *Speciality: clinical areas managed*.

d *Languages: network languages*.

e *Sponsor: country of sponsor*.

f *Catchment: countries of referrals*.

g *Activity: 0 = no activity (fewer than 10 clinical cases); 1 = some cases handled but not active at the time of study; 2 = active at the time of study*.

**Figure 2:**
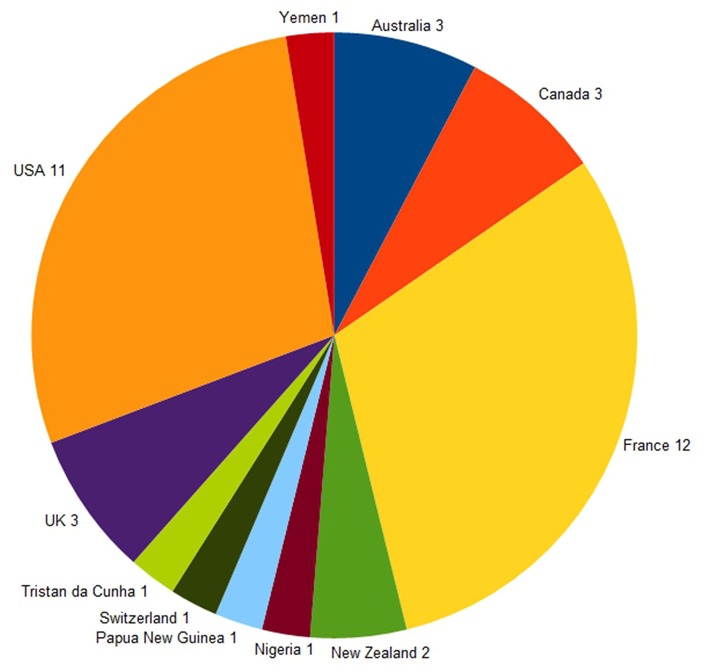
Countries of the network sponsors (*n* = 39).

The networks were mainly set up by informal groups of clinicians (30 of the 39 networks). The sponsor of each network (i.e., the person requesting that it be established) was most commonly based in France or in the US (12 and 11 networks, respectively, see Figure 2). All networks which stated the fact in their prospectus were using volunteer specialists to provide the necessary expertise.

Four networks were established by charitable organizations and five networks were established by other organizations (e.g., those investigating telemedicine for commercial or semi-commercial reasons).

Approximately half of the networks were set up to use a single language, most commonly English. However, the Collegium system is available in four other languages (French, Spanish, Portuguese, and Arabic) and almost half of the networks elected to work in multiple languages (see Table 3).

**Table 3:** Languages used by the clinical and educational networks.

Languages*	**No of networks**
English	20
English/Arabic	2
English/French	10
English/French/Spanish	2
English/French/Spanish/Portuguese	1
English/Spanish	3
French/English	1
Total	39

* *Primary network language shown first*.

### Activity

There was wide variation in the activity levels on the networks. Of the 39 potentially operational networks which were set up (i.e., those established for clinical or educational purposes), 15 networks (38%) were stillborn and had not handled a single case after being established. In contrast, the two most active networks, both clinical, had handled almost 12,000 cases.

A total of 33 networks had been set up to provide a clinical service. All but one had been established to provide a service to referrers in low-resource settings, mainly in developing countries; one network was operating in an industrialized country (albeit one with referrers based in remote regions). Six of the 33 clinical networks (18%) could be considered to have matured into routine services, having handled more than 100 cases each (see Table 4). Two of the six educational networks (33%) could be considered to have matured into routine services, having handled more than 100 cases each.

**Table 4:** Networks which had handled more than 100 cases (excluding administrative/test networks).

**Network ID**	**Purpose**	**Duration of operation (days)**	Total no of cases*	**Mean case rate (cases/week)**
14	Educational	2016	165	0.6
18	Clinical (not low resource setting)	1966	2697	9.6
22	Clinical	2191	9210	29.4
25	Clinical	1643	686	2.9
26	Clinical	1609	121	0.5
34	Educational	926	217	1.6
42	Clinical	639	449	4.9
45	Clinical	449	123	1.9

* *Excluding test cases*.

**Figure 3:**
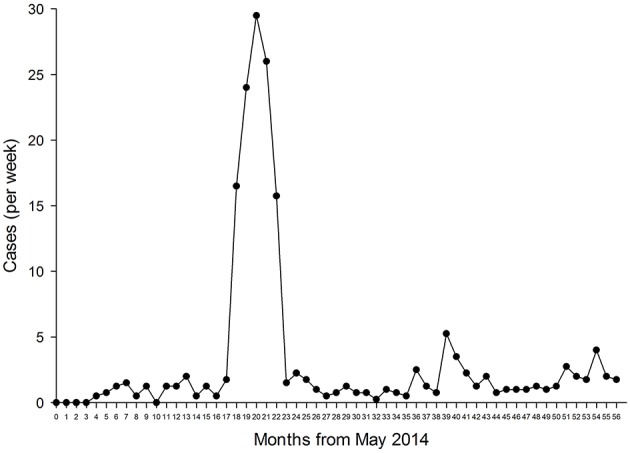
Weekly case rate in network 25.

The average case rate of the five clinically-active networks operating in low-resource settings (i.e., the total number of cases divided by the length of time for which the network had been established) ranged from 0.5 to 29.4 cases/week. However, within the networks, the case rates fluctuated considerably. For example, approximately 2 years after it had been established network 25 demonstrated a large peak in its the weekly case rate (see Figure 3). This peak was due to cases being submitted from a hospital which suffered a sudden shortage of specialists. When new staff were appointed at that hospital, its use of telemedicine ended abruptly; the network reverted to a relatively constant level of referrals, as demonstrated across the remainder of the study period.

**Figure 4:**
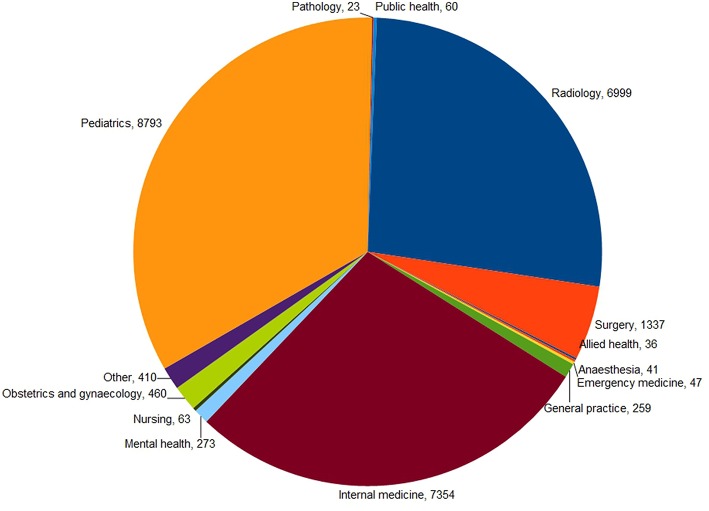
Types of queries resulting from cases that were managed in 39 non-test networks (*n* = 26,155).

There was a wide range of types of case handled in the 39 non-test networks, including medical, surgical, nursing, and allied health (Figure 4).

### Survey

The survey was sent to 49 network coordinators, from 31 networks. Responses were received from 9 coordinators (18% of those invited to participate; Table 5). The median satisfaction score was 8. The comments made by the responders mainly concerned the technical aspects of the system. Overall, they stated that they were satisfied with it and grateful to be able to use a system that was reliable and efficient. The free-text comments are summarized in Table 6.

**Table 5:** Summary of survey responses.

**Network ID**	**Active network?**	**Type**		**Overall satisfaction**	**Recommend to others?**	**Specific staff?**	**(1) Suggestions?**	**(2) Main benefits?**	**(3) Main difficulties?**	**If not using, why?**	**(4) Further comments?**
36	N	(Clinical)		10	Y	N	N			Y	
33		Clinical		8	Y	N	Y	Y	Y		Y
25		Clinical		10	Y	N	N	Y	Y		Y
42		Clinical		7	Y	?	Y	Y	Y		Y
25		Clinical		8	“depends”	N	Y	Y	Y		Y
60	N	(Clinical)		8	Y	N	Y	Y	Y	Y	Y
14	N	(Educational)		8.5	Y	N	Y	Y	Y	Y	Y
34		Educational		7	Y	N	Y	Y	Y		Y
50	N	(Educational)		10	Y	N	N			Y	Y
			**Median**	**8**							

**Table 6:** Free-text responses to survey.

(1) Suggestions	secure text messaging feature, i.e., being able to consult via text message (2) integrate with a real-time voice over internet system, such as WhatsApp talk in real time improve app speed can be used offline, available (such as with Dropbox)
(2) Main benefits	storage/record keeping (4) secure, confidential (3) Quality Assurance options (2) Coordinator following up cases free (no fee charged) platform (better than the use of email) accessibility easy to use simple and intuitive (no training necessary) robust access to expertise where there is no specialist
(3) Main difficulties	internet connectivity (2) DICOM image transfer legislation compliance mobile application too slow (delay after login) mobile app not user friendly need to remember username and password not accessible through text message user account inactivated after non-use for a while data storage security possible ownership issue
(4) Further comments	thank you and positive comments for the effort done (5) issue with telemedicine (lack of patient feedback, back and forth communication, considering expert access value)

## Discussion

Our aim in making the Collegium system available was to facilitate the introduction of telemedicine by organizations delivering health care in low-resource settings. There is reasonable evidence that telemedicine in this environment is clinically useful and provides valuable support for remote staff (3, 4). However, a barrier to any organization contemplating the introduction of a telemedicine service is the provision of the necessary technical infrastructure. Unless the organization is able to join an existing network operated by somebody else, then it will be necessary to start one from scratch, either using existing software or building the software required. While starting a telemedicine network from scratch is perfectly possible, it requires appropriate technical expertise. Also, an underlying IT infrastructure is required, e.g., a web server, whether existing software or bespoke software is employed. The point of the Collegium approach is that both the software and the IT infrastructure are made available as an integrated package, so that only minimal setting up is required before telemedicine work can begin.

However, the technical infrastructure is not the only thing required in order to establish a successful and sustainable telemedicine service. That is, the infrastructure is a necessary but not sufficient condition for successful telemedicine (as was pointed out by one of the survey respondents), because there must also be engagement from referrers submitting cases, experts who provide responses, and some kind of clinical supervision to ensure that the whole process runs smoothly. These human factors are critical to the initial phases of a telemedicine network, and to its eventual adoption into routine health care (or not).

During the first 6 years of operation, 33 networks were set up to provide a clinical telemedicine service. This verifies our hypothesis that organizations delivering health care in low-resource settings would use the Collegium system to establish trial telemedicine services. Of these, six networks could be considered to have matured into routine clinical services, i.e., they could be considered as successful networks from the Collegium point of view. There are no published data from any similar system to compare this with, and we are not able to conclude that a “success” rate of 18% is either disappointing or encouraging. We are able to state however, that many of the “failed” networks did not process a single telemedicine case, i.e., failure occurred at the initial inception, rather than after the first few months when the workload began to build up. We can also speculate that the networks with zero activity may have prevented their parent organizations from wasting scarce resources on setting up expensive telemedicine networks that were then unused. In this sense, perhaps these networks can be counted as a success.

Wide variations of activity were observed in the 39 clinical and educational networks established over the 6-year study period. We do not know the reasons for this, but we can assume that it is related to the non-infrastructural factors necessary for successful telemedicine:

1. a Referrer who wants to seek an outside opinion on a patient and is sufficiently motivated to do so
2. a mechanism to connect this Referrer to an appropriate Specialist
3. a Specialist who can provide the necessary expertise.

Practical experience shows that the mechanism that matches demand for information to supply can only be partially automated; some human intervention is almost always required. In the Collegium system, this is provided by one or more network Coordinators whose job is to supervise the case flow, and when necessary to select suitable Specialists for the problem in hand. Although some of the successful networks have exhibited signs of strain in managing high case loads, we are not aware that any network has failed because of a lack of human Coordinators. Nor do there seem to have been problems in recruiting appropriate Specialists. We suspect that many of the networks which failed to establish themselves failed at point 1, i.e., they were unrealistic in expecting referring doctors to submit cases.

The underlying reasons for unrealistic expectations about initial referrals in a new network are not known. One possibility may be that local doctors practise in an environment where access to specialist opinions is always difficult, so they are used to managing complex cases alone, and there is no custom and practice of obtaining external advice. Another possibility is that they are working in a healthcare system that is so damaged or non-existent, that priorities are different and push local doctors to manage patients by themselves, without any expert advice. These are all factors identified in Labrique et al.'s review of best practice for scaling digital health initiatives in LMICs which were derived from practical experience in real-life case studies (5). They represent an important area for future research.

### Growth of Activity

**Figure 5:**
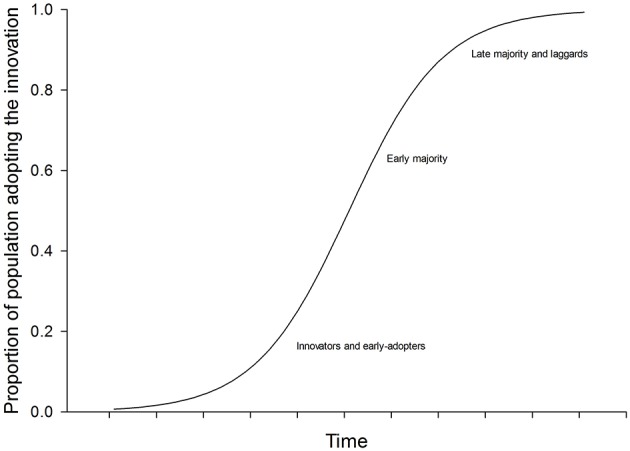
Sigmoidal population growth.

The networks using the Collegium system had mainly come to it *ab initio*, i.e., they wished to start a new telemedicine network within their sphere of operations where none had existed before. What pattern of network activity would therefore be expected? Clearly activity would begin from zero, and would, if the network were successful, eventually settle at some reasonably steady rate which would reflect the routine use of telemedicine in their health care environment. It would not be unreasonable to expect a sigmoidal growth curve (Figure 5). Such growth curves are widely observed as technological innovations are adopted in health care, and into industry more generally. Growth curves of this shape result from the gradual adoption of a new technique within an organization during which early adopters accept it quickly, while late adopters (“laggards”) take much longer (6).

**Figure 6:**
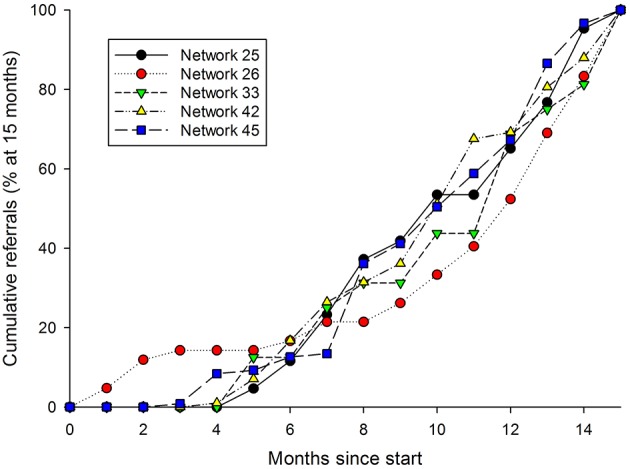
Cumulative referral rates from five networks, during the first 18 months of their operation.

It is not clear, however, whether sigmoidal growth curves can be expected in telemedicine. Grigsby et al. provided one of the first quantitative reports on the adoption of telemedicine in North America (7). However, the data they cited on the numbers of teleconsultations reported during consecutive annual surveys show little evidence of sigmoidal growth. Similarly, a later study by Darkins provided little evidence of sigmoidal growth in the number of teleconsultations in the VHA healthcare system (8). The Collegium data are consistent with these reports. Taking the five small clinical networks and calculating their referral “trajectories” (i.e., adjusting their cumulative referrals so that they all start at month zero and end at 100% after 15 months) shows that there is only weak evidence of accelerating growth after start-up (Figure 6).

## Conclusion

During the first 6 years of the availability of the Collegium system, a total of 39 telemedicine networks were set up for clinical or educational purposes. Although a substantial proportion of the networks that were set up using the Collegium infrastructure—approximately one-third—did not handle a single clinical case, this might represent a form of success in the sense that it prevented the waste of resource involved in an organization purchasing a telemedicine infrastructure only to find that it was not used. The reasons for unrealistic expectations about initial referrals in a new network are not presently understood.

The remaining 62% of networks handled a wide range of clinical cases, and at activity levels ranging from less than one case per week to several cases per day. The overall satisfaction of the network coordinators who responded to the survey was uniformly high. They identified many benefits in using the system and offered various constructive suggestions about improving its future development.

The present study suggests that the Collegium system has fulfilled its aims in providing useful support for a range of organizations conducting humanitarian or non-commercial work in low resource settings. However, it only solves part of the problem of setting up a successful telemedicine network, and organizations contemplating such a step should not underestimate the non-technical aspects.

## Data Availability

All datasets generated for this study are included in the manuscript and/or the Supplementary Files.

## Author Contributions

RW and LB conceived and designed the study, contributed to writing the manuscript, and approved the submitted version.

### Conflict of Interest Statement

The authors declare that the research was conducted in the absence of any commercial or financial relationships that could be construed as a potential conflict of interest. The authors are members of the Steering Group for Collegium Telemedicus, which is a not-for-profit organization that makes the Collegium telemedicine system freely available for humanitarian purposes (see https://collegiumtelemedicus.org).

## Footnotes

^1^Collegium Telemedicus home page. Available online at: https://collegiumtelemedicus.org
