# Supplementary material for: Experience of Supporting Telemedicine Networks With the Collegium System: First 6 Years
Source: Front Public Health. 2019 Aug 21;7:226. doi: 10.3389/fpubh.2019.00226 (PMC6712066; doi:10.3389/fpubh.2019.00226)
Supplement: Supplementary file 1 [file Data_Sheet_1.docx]

**Appendix 1. Survey**

1.What is your overall satisfaction with the Collegium system? from 1 (not at all satisfied) to 10 (very satisfied)

2. Would you recommend it to other potential users? Y/N

3. Are there any staff specifically employed to manage your telemedicine network? Y/N

4. Do you have any suggestions for improving the system?

5. If you are currently using your network-

5a. What are the main benefits from your point of view?

5b. What are the main difficulties in using it?

6. If you are NOT using your network-

6a. Why not?

7. Do you have any further comments?
